# Veterans Health Administration nurses’ training and beliefs related to care of patients with traumatic brain injury

**DOI:** 10.1371/journal.pone.0222585

**Published:** 2019-09-16

**Authors:** Tolu O. Oyesanya

**Affiliations:** Duke University School of Nursing, Durham, NC, United States of America; Monash University, AUSTRALIA

## Abstract

**Background:**

Veteran patients with traumatic brain injury (TBI) and their family members regularly receive care from nurses. Understanding nurses’ training and beliefs can provide direction for intervention work aimed at ensuring the best possible care is delivered to this population.

**Aims:**

We examined Veterans Health Administration (VHA) nurses’ training and beliefs related to care of patients with moderate-to-severe TBI.

**Design and methods:**

We conducted an exploratory, cross-sectional survey with 211 VHA nurses and analyzed data using descriptive statistics.

**Results:**

The average years of nursing experience was 18 years, and 90% reported ever caring for a patient with TBI. Most nurses (70%) reported only seeing patients with TBI ≤1–2 times per year in their current role; 20% reported seeing these patients 1–2 times per month. Even with infrequent care, almost 50% reported previously receiving TBI-related training. Beliefs items with the highest accuracy indicated nurses agreed that they need specialized training to care for patients with TBI and that TBI recovery may continue for several years (96.39% accuracy respectively). The beliefs item with the lowest accuracy indicated focus on whether nurses agreed that TBI severity was important in developing care plans (27.84% accuracy). Nurses reported the need for clarity of the nursing role in caring for patients with TBI (77.32% agreement).

**Conclusion:**

VHA nurses do have accurate beliefs about caring for Veteran patients with moderate-to-severe TBI; however, there is the need for further role clarification regarding nursing care of patients with TBI.

**Impact:**

These findings have implications for development of education and training interventions for nurses who care for Veteran patients with TBI.

## Introduction

Traumatic brain injury (TBI) is the signature injury for Veterans returning from recent wars [1], with over 333,000 Veterans sustaining a TBI worldwide since 2000. TBI still occurs frequently among Veterans, with over 25,000 Veterans diagnosed with TBI annually between 2010 and 2014 [2]. TBI is a chronic disease that causes immediate and lifelong problems for those who sustain the injury [3], which has significant implications for how nurses provide care for these patients.

## Background

### Traumatic brain injury

TBI can be characterized as mild, moderate, or severe, and the differing severity levels cause predictably different impairments [4]. Mild TBI is known to cause poor physical functioning, including fatigue and emotional distress, which may resolve in a few weeks [5]. Although mild TBI has higher incidence rates compared to moderate-to-severe TBI [6], a large portion of care for Veteran patients with mild TBI occurs in an outpatient setting [7], as over one-third of mild TBIs go undiagnosed and untreated [8]. The influence of the above-listed cognitive impairments associated with mild TBI are debated in the literature, but are often limited [4]. As Veteran patients with mild TBI typically have a full recovery [8], mild TBI is not the focus of this paper.

Moderate-to-severe TBI causes more marked impairments that are immediate and long-lasting, including significant problems with communication skills, psychosocial and emotional distress, and problems with attention, memory, concentration, and executive function [9]. These impairments influence the Veteran patient’s ability to communicate effectively, learn new information, and decrease their speed of information processing. As the effects of moderate-to-severe TBI have a marked lifelong influence on the lives of Veteran patients with TBI and their families [3], moderate-to-severe TBI is the focus of this paper.

Immediately after sustaining a moderate-to-severe TBI, patients who survive the initial injury are referred to as having an *acute moderate-to-severe TBI*, beginning the acute phase of recovery. The *acute phase of TBI* is defined as patients who survived an acute moderate-to-severe TBI and are seeking critical care and treatment to minimize secondary injury and receive life support and rehabilitation [10]. The acute phase of TBI can lasts up to six months after the initial injury and usually ends at hospital discharge [10]. Typically, after discharge from the hospital, the chronic phase of recovery begins. The *chronic phase of TBI* encompasses patients with *chronic moderate-to-severe TBI* (i.e., patients with a history of moderate-to-severe TBI) who deal with TBI as a lifelong health condition, particularly because TBI “impairs the brain and other organ systems and may persist or progress over an individual’s life span” [3, p. 1199]. The chronic phase of TBI lasts from hospital discharge for the remainder of the patient’s life span [3]. As many Veterans age 18 to 24 years have sustained a moderate or severe TBI [1], these impairments will influence many aspects of their life during both the acute and chronic phase of TBI recovery, including when seeking care for other health conditions later in life [3].

### Care pathways after sustaining a moderate-to-severe TBI

Veterans can sustain a moderate or severe TBI in multiple ways, including in combat and during common daily activities [1]. Depending on the location where the TBI was sustained (e.g., in military theater, on military base, at home), Veterans who sustain an acute moderate-to-severe TBI may receive immediate or subsequent care at Veterans Health Administration (VHA) hospitals for acute TBI treatment and rehabilitation. Veterans may also receive care at different phases of recovery, including during the chronic phase of TBI recovery [11], where many Veterans also seek care at VHA hospitals or non-VHA hospitals for other health conditions related or unrelated to their TBI [12].

In fact, in 2005, the VHA developed an integrated nationwide Polytrauma System of Care (PSC) that provides cutting edge rehabilitation services and ensures Veterans and service members with polytrauma (including TBI) smoothly transition between the Department of Defense (DoD) and VHA, and back to their own communities. This network of over 100 programs specializes in clinical rehabilitation services and is organized across four levels of care.

- **Level 1: Polytrauma Rehabilitation Center (PRC)**. The PRCs serve as regional referral centers for the comprehensive acute rehabilitation for complex and severe polytrauma.
- **Level 2: Polytrauma Network Site (PNS).** A PNS is located in at least 1 VA medical center in each Veterans Integrated Service Networks (VISN). The PNS provides post-acute inpatient and outpatient rehabilitation care and coordinates polytrauma and TBI services throughout the VISN.
- **Level 3: Polytrauma Support Clinic Team (PSCT).** PSCTs provide services and organize key components of outpatient post-acute rehabilitation care for polytrauma and TBI within the facility’s geographical area. PSCTs also conduct comprehensive assessments of patients who are confirmed positive for TBI and develop and implement rehabilitation and community reintegration plans.
- **Level 4: Polytrauma Point of Contact (PPOC).** The outpatient PPOC ensures that those with polytrauma and TBI, who would benefit from rehabilitation, are referred to the appropriate PSC programs or local VA and community specialty care resources.

A data extraction from the Veterans Informatics Computing Infrastructure (VINCI) showed, until June 2016, a total of 441,223 Veterans from recent wars have received TBI care during the acute or chronic phase of TBI at a VHA hospital in the United States [13]. Between June 1, 2015 and June 1, 2016, approximately 50,240 Veterans received care for a moderate or severe TBI during the acute or chronic phase of TBI recovery at a VHA hospital in the U.S. [13]. Of these Veterans, 14,118 received care for a moderate or severe TBI during the acute phase of TBI, while 17,898 received care during the chronic phase of TBI [13]. This data provides evidence that the majority of TBI care provided at VHA hospitals in the U.S. is to Veterans during the chronic phase of TBI (i.e., Veterans with a history of moderate-to-severe TBI). As most Veteran patients with TBI are receiving care at VHA hospitals in the U.S. during the chronic phase of TBI, it is important that all VHA nurses who provide care to these patients are knowledgeable about caring for these patients.

### Research on nurses’ beliefs and training related to care of patients with TBI

Limited literature is available that assesses nurses’ beliefs or TBI-related training specific to care of Veteran patients with moderate-to-severe TBI. To-date, research on nurses’ beliefs mostly focuses on patients with mild TBI, showing that nurses have inaccurate beliefs, also known as perceptions, about caring for these patients [14], including in a mixed sample of VHA and non-VHA nurses [15]. Among nurses, there are inaccurate beliefs about the following topics related to patients with TBI including: use of seatbelts when injured, unconsciousness, impairments caused by TBI, and recovery after TBI [14]; sex-based differences and the use of medical labels [16]; and negative attitudes towards patients with TBI, including use of visibility of injury as an indicator of how much care a patient should receive [17], [18]. In addition, research has also shown that approximately 76% of nurses who care for Veterans patients with combat-related TBI injuries (severity levels unspecified) perceive these patients over report pain intensity [19]. Overall, nurses’ beliefs about care of patients with moderate-to-severe TBI are important to assess because providers’ beliefs can influence their own practice behaviors [20] and because presence of inaccurate beliefs can cause nurses to provide inaccurate information to patients and families [14].

In addition, research shows nurses need more education and training to care for patients with moderate-to-severe TBI. Nurses have reported that their prior education and training was insufficient to care for the patients with brain injuries [21], [22]. Nurses have also emphasized the need for specialized education and training to care for patients with TBI [16] and patients with other neurological conditions [21], [22]. In particularly, 91% of nurses reported that specific training and education to care for patients with neurological conditions, such as TBI, be mandatory [21].

Finally, research shows multiple factors can influence the care nurses provide to patients with TBI, suggesting care of these patients needs to be standardized to prevent variation from institution to institution [23] or even nurse to nurse. Variables that could cause variations in care of patients with moderate-to-severe TBI include nurses’: 1) beliefs related to care of this population [16]; 2) TBI-related training [24]; 3) experience caring for patients with TBI [24]; and 4) focus on caring for patients with moderate-to-severe TBI in the acute phase of recovery rather than the chronic phase [25].

### Clinical guidelines on nursing management of patients with TBI

Similar to the limited research for nurses to use to direct their care, there are gaps in evidence-based clinical guidelines for non-acute nursing management of patients with moderate-to-severe TBI. Multiple interdisciplinary guidelines have been published on care of patients with mild, moderate, or severe TBI, including in the areas of critical care or polytrauma [26–30]. However, it is not clear if nurses are aware of these guidelines or if they are using them in their current practice. In addition, there are no nursing-specific guidelines available that provide nurses with direction in areas such as: 1) the influence of cognitive impairments on communication with and education of patients with acute or chronic moderate-to-severe TBI, 2) sex-based differences in recovery after acute and chronic moderate-to-severe TBI, or 3) non-acute nursing management of patients with chronic moderate-to-severe TBI.

The dearth of literature on nurses’ beliefs and TBI-related training specific to care of these patients and lack of clinical guidelines on non-acute nursing management provide limited direction for nurses to use to guide the care they provide. Thus, more research is needed to identify areas where additional guidance is needed to direct the development and testing of education and training interventions for VHA nurses who care for Veteran patients with TBI. To this end, the purpose of this study was to examine VHA nurses’ TBI-related training and beliefs about caring for Veteran patients with moderate-to-severe TBI.

## Methods

### Design & data collection

We conducted this study using a cross-sectional, exploratory design. Participants were recruited from two VHA hospitals in the Midwestern region of the United States. Both VHA Hospitals were PSC Level 3 (Polytrauma Support Clinic Team- PSCT), which means they do not provide care in the acute phase following a TBI but rather provide care coordination services with key components of rehabilitation care for polytrauma and TBI within the facility’s geographical area. The methods and findings in this paper are reported in accordance with STROBE guidelines (see S1 STROBE Checklist) [31].

### Participants

Data were collected between June and August 2015. All registered nurses employed by the participating hospitals (n = 1,381) were invited to participate via email invitation containing a link to the electronic survey. Nurses across all hospital departments were asked to participate because all VHA nurses may care for patients with acute or chronic moderate-to-severe TBI depending on severity of injury, time since injury, and comorbidities [32]. Nurses were eligible to participate if they were registered nurses and employed by the participating hospitals and outpatient clinics.

### Ethical considerations

This study was approved by the University of Wisconsin-Madison Health Sciences institutional review board and VA Research & Development Committee (IRB #2014–1602), including obtaining a waiver of written consent. When participants began the electronic survey, the first page detailed the informed consent with a request that participants read the page in its entirety before proceeding. The informed consent notified participants that their participation was anonymous and voluntary, including a statement that they could stop answering questions at any time by exiting their browser. The study purpose, risks and benefits of participation, and data management plans were also described. The informed consent page ended with the following statement: “After you have read this consent, you may proceed to the survey on the next page by clicking on ‘Continue.’ Completing the survey implies consent to participate in the study. You make leave the survey at any time by closing your web browser.”

### Measures

#### Demographics

Nurses answered demographic items, including age, sex, highest nursing degree, years of active nursing practice, years at current position, primary role, primary work setting, and age of patients seen.

#### Perceptions of brain injury survey

As part of a larger study, we used a modified version of The Perceptions of Brain Injury Survey (PBIS), which contains independent subscales that can be used to assess nurses’ TBI-related training and beliefs related to care for patients with moderate-to-severe TBI. Detailed information on survey development [16] and reliability and validity [33] are published elsewhere. Cronbach’s alpha (r) and composite reliabilities (ω) also known as McDonald’s omega coefficient] are reported for the TBI-related training and beliefs subscales as composite reliabilities are known to give a better reliability estimate [34]. McDonald’s omega coefficient is a reliability measure that indicates whether items measure the same latent variable [34].

#### TBI-related training

Eleven items assessed nurses’ prior TBI-related training and clinical experience. These items assessed if nurses’ clinical practice had ever included patients with moderate-to-severe TBI, TBI-related training history, types of training, and frequency of providing care to patients with TBI. Item formats included likert scale, multiple choice, yes/no, and check-all-that-apply (r = 0.79; ω = 0.89).

#### Beliefs

There were 17 items that assessed nurses’ beliefs about caring for patients with moderate-to-severe TBI, including topics such as: care provision, incidence rates, sex-based differences, recovery, and the role of nurses in caring for this population. Items were scored on a 4-point Likert-type scale from strongly agree (1), agree (2), disagree (3), and strongly disagree (4) (r = 0.62; ω = 0.92).

### Data analysis

#### Missingness

We tested for missingness prior to conducting statistical analyses. Using Stata, we determined data were not missing at random or completely at random [35]. Percentage of missingness by subscale ranged from 11.5 to 22.3%. Next, we imputed partially missing data for each survey domain with multiple imputation using chain equations (MICE) [36].

#### Descriptive statistics

Means, frequencies, and percentages were calculated to describe nurses’ overall demographics, TBI-related training, and beliefs. For TBI-related training, frequency of care items were scored on a 3-point Likert scale: ≤ 1–2 times per year (1), 1–2 times per month (2), and ≥ 1–2 per week (3). All other items were dichotomous [yes (1), no (0)]. Beliefs items were initially scored on a 4-point Likert scale [strongly agree (1), agree (2), disagree (3), and strongly disagree (4)]. When computing percentages, agree and disagree variables were collapsed for ease of interpretation (i.e., responses marked strongly agree (1) and agree (2) were collapsed and reported as ‘agree’). Results on TBI-related training and beliefs are listed in rank order based on percentage.

## Results

### Sample characteristics

A total of 211 VHA nurses completed the survey, a 15.27% response rate. According to Shih and Fan (2009), response rates in electronic surveys are typically 20% or lower, thus our response rate is typical [37]. Most VHA nurses who participated were women (87%), middle aged (mean 45 years), held a bachelor’s degree in nursing (56%), and worked as a staff registered nurse (71%). Although one-third of nurses (37%) worked on an inpatient unit, most nurses (40%) reported their work setting as “other” (including operating room, medical sub-specialty, and a psychiatric unit), and 10% reported working in a primary care setting. Most nurses provided care to older patients, with 80% of nurses reporting they see patients age 60 years or older. Participant characteristics are described in Table 1.

**Table 1:** Participant characteristics (n = 211).

Demographic Variables	mean (SD)
Age, mean years (SD)	45.79 (12.24)
Total years in active nursing practice, mean y (SD)	18.11 (13.05)
Total years in current nursing position, mean y (SD)	6.09 (6.85)
Years since graduate from highest nursing degree	13.63 (19.03)
	n (%)
Sex	
Female	184 (87.20)
Male	27 (12.80)
Nursing education	
Associates’, Nursing Diploma, Other	38 (18.01)
Bachelors’	120 (56.87)
Masters’ or Doctorate	53 (25.12)
Primary Work Setting	
Primary Care Clinic	23 (10.90)
Emergency Room	8 (3.79)
Ambulatory Clinic or Doctor’s Office	17 (8.06)
Inpatient Unit	80 (37.91)
Other	86 (40.76)
Primary Role	
Staff Registered Nurse	150 (71.09)
Nurse Practitioner/Clinic Nurse Specialist/CRNA	14 (6.64)
Division Officer/Charge RN/Care Team Leader	4 (1.90)
Department Head/RN Supervisor/Nurse Manager	13 (6.16)
Other	30 (14.22)
Age of patients seen	
13 to 18 years	1.42% (0.11)
19 to 39 years	61.13% (0.48)
40 to 59 years	71.09% (0.45)
60 years and older	80.56% (0.39)
Does not see patients	16.58% (0.37)

Note: CRNA = Certified Registered Nurse Anesthetist; RN = Registered Nurse

### TBI-related training

In this highly experienced sample of nurses (average 18 years’ experience), 90% reported ever having cared for a patient with moderate-to-severe TBI. However, the majority of nurses (70%) reported only seeing patients with moderate-to-severe TBI ≤1–2 times per year in their current role; another 20% reported seeing these patients 1–2 times per month. Even with the low frequency of care of patients with moderate-to-severe TBI, almost 50% of nurses reported having received TBI-related training at some point in their career. Of the 105 nurses who had TBI-related training, 44% received training in the form of in-services, workshops, conferences, or seminars. Regarding location of training, most VHA nurses with TBI-related training reported receiving training at poster sessions, technical sessions, mini seminars, or short in-services. Only 1.42% of nurses with TBI-related training received their training as part of a college course devoted primarily to the topic of TBI and its consequences (see Table 2).

**Table 2:** Percent values for TBI-related training items (n = 211).

TBI-Related Training Items					Selected (Yes)	
					n	%
Has your clinical practice ever included patients with TBI?					188	89.09
Have you had specific training related to TBI?					105	49.76
Was the specific training part of…						
Undergraduate or graduate education					15	7.10
In-services, workshops, conferences, seminars, or other forms of continuing education					93	44.07
Other					18	8.53
Which categories best describe where you received your training related to TBI? (Check all that apply)						
Poster sessions, technical sessions, mini seminars, or short in services					60	28.43
Half-day or full-day conferences such as short courses, seminars, or workshops					31	14.69
University courses taken after completion of your degree					6	2.84
Other					34	16.11
Did you take one or more college courses devoted primarily to the topic of TBI and its consequences?					3	1.42

Frequency of Caring for Patients with TBI	≤ 1-2x / year		1-2x / month		≥ 1-2x / week	
	n	%	n	%	n	%
In your current position, how often do you see patients with moderate-to-severe TBI?	155	73.45	44	20.85	12	5.68
In your current position, how often do you see MEN with moderate-to-severe TBI?	154	72.98	47	22.27	10	4.73
In your current position, how often do you see WOMEN with moderate-to-severe TBI?	208	98.57	3	1.43	0	0

### Beliefs

Nurses were asked to rate their agreement or disagreement with statements on various topics related to care of patients with TBI (see Table 3). More than 92% of nurses accurately agreed with the statements: 1) “To assess and/or treat patients with TBI, nurses need specialized TBI educational materials”; and 2) “Knowing the location of brain damage from TBI helps in the development of nursing care plans that meet patients’ needs.” Regarding sex-based differences after TBI, over 78% of VHA nurses in our sample accurately disagreed with the statements: 1) “TBI is equally common in men and women”; 2) “Men and women recover in the same way after having a TBI”; and 3) “Family involvement in patient care is no different for men and women who are receiving care after having a TBI.” Over 72% of nurses inaccurately agreed with the statement: “Medical labels that specify TBI as mild, moderate, or severe are useful for development of nursing care plans.” Over 80% of nurses accurately agreed with the statement: “Knowing about moderate-to-severe TBI is important to my current nursing practice.” Although 71% of nurses agreed with the subjective statement, “Nurses on my unit do a good job when providing care to patients with moderate-to-severe TBI,” over 77% of nurses disagreed with the subjective statement: “The role of registered nurses in regard to care of patients with TBI is clearly understood in my workplace.”

**Table 3:** Percent values of beliefs items.

Please rate your agreement or disagreement with the following statements.	Accurate/ Inaccurate	Agree		Disagree	
		n	%	n	%
1. Nurses need specialized training to provide care to patients with moderate-to-severe TBI.	Accurate	187	96.39	4	3.61
2. Recovery following TBI may continue for several years.	Accurate	187	96.39	4	3.61
3. Knowing the location of brain damage from TBI helps in the development of nursing care plans that meet patients’ needs.	Accurate	185	95.36	6	4.64
4. To assess and/or treat patients with TBI, nurses need specialized TBI educational materials.	Accurate	180	92.78	11	7.22
5. Knowing about moderate-to-severe TBI is important to my current nursing practice.	Accurate	161	82.98	30	17.02
6. Nursing care plan goals for patients with TBI may need to be revised more frequently than nursing care plan goals for patients with other types of disabilities.	Accurate	151	77.83	40	22.17
7. Greater variability exists in the population of patients with TBI than exists in populations of other patients with disabilities.	Accurate	150	77.31	41	22.69
8. The challenges of patients with TBI are typically more difficult to assess than the challenges of patients with other disabilities.	Accurate	147	75.77	44	24.23
9. Men and women require different types of care after having a TBI.	Accurate	145	74.74	46	25.26
10. Medical labels that specify TBI as mild, moderate, or severe are useful for development of nursing care plans.	Inaccurate	140	72.16	51	27.84
11. Nurses on my unit do a good job when providing care to patients with moderate-to-severe TBI.	N/A*	139	71.64	52	28.36
12. Patients with TBI often display behavior problems.	Accurate	135	69.58	56	30.42
13. Family involvement in patient care is no different for men and women who are receiving care after having a TBI.	Inaccurate	55	28.35	136	71.65
14. The role of registered nurses in regard to care of patients with TBI is clearly understood in my workplace.	N/A*	44	22.68	147	77.32
15. TBI is equally common in males and females.	Inaccurate	42	21.64	149	78.35
16. Knowledge of a patient’s background prior to TBI is necessary when developing a nursing care plan.	Accurate	33	17.01	158	82.99
17. Men and women recover in the same way after having a TBI.	Inaccurate	25	12.88	166	87.12

Note: N/A* indicates a subjective item based on the beliefs about nurses’ performance in the workplace and cannot be rated as an accurate or inaccurate belief.

## Discussion

The purpose of this study was to examine VHA nurses’ TBI-related training and beliefs related to care of Veteran patients with moderate-to-severe TBI. Findings showed that VHA nurses had varying levels of TBI-related training and beliefs about care of patients with TBI. Although most of our sample reported their clinical practice has ever included patients with moderate-to-severe TBI, few nurses cared for these patients regularly. Even with low frequencies of caring for patients with moderate-to-severe TBI, most VHA nurses had majority accurate beliefs related to care of patients with TBI.

In our highly experienced sample (average 18 years of experience), 90% of the nurses reported having ever cared for a patient with TBI at some point in their career. However, more than 70% of nurses in the sample rarely saw patients with moderate-to-severe TBI, reporting a frequency of care of ≤1–2 times per year. Although some may question whether nurses in our sample were aware that they may be providing care to patients with chronic moderate-to-severe TBI, this report of low frequency of care was likely due the classification of the VHA hospitals where we recruited. The nurses in our sample were recruited from two PSC Level 3 (Polytrauma Support Clinic Team- PSCT) VHA hospitals, which means these hospitals do not care for Veteran patients with acute TBI but rather provide services and organize key components of rehabilitation care for polytrauma and TBI within the facility’s geographical area. These facilities have careful TBI screening mechanisms in place in the outpatient setting to identify patients with a history of TBI. When a positive TBI screen occurs, the patient is seen in the specialized TBI clinic. Therefore, the low frequency of care is likely due to the fact that the general sample of nurses at these recruitment sites may not see as many patients with TBI because of the highly specialized care this population receives with in the VHA, not because these nurses are unaware of the history of the patient. In addition, of those with experience caring for patients with TBI, less than half indicated they had prior TBI-related training. The fact that 50% of the sample has not received specific TBI-related training may be due to the low frequency of care of patients with TBI at these recruitment hospitals. However, it is important to note that for nearly a decade, a one-time educational training course on TBI has been required for all clinical employees at VHA hospitals, including nursing staff [38].

Our findings also indicate that although nurses had mostly accurate beliefs, there are some discrepancies in nurses’ beliefs about caring for patients with TBI. More than 70% of the sample inaccurately agreed with the statement “Medical labels that specify TBI as mild, moderate, or severe are useful for development of nursing care plans.” This belief is inaccurate because use of specifications about severity of injury alone are not enough to provide direction for development of the patient’s plan of care [39]. Each TBI is quite unique; two people who sustain a moderate TBI in the same region of the brain could have heterogeneous outcomes with different deficits, impairments, prognoses, and optimal points of recovery [39]. Unfortunately, there is limited diagnostic precision with tools used to assess severity of TBI (such as the Glasgow Coma Scale), so sole use of medical labels (such as those that specify severity of injury) to develop a plan of care is not sufficient [39]. While medical labels about the severity of injury may be useful in understanding the patient’s needs on a global level, these labels are not enough to develop a care plan; nurses also need results of patient performance on numerous neuropsychological, cognitive, and motor assessments to use in developing and directing the patient’s care plan (including therapy integration and therapy follow through) to ensure all impairments and needs are appropriately addressed [39], [40]. However, this item on use of medical labels may have had issues with participants’ interpretation of the meaning of the question vs. preferred response; there may be a false equivalency between the phrasing of the question (“useful”) and the interpretation of the preferred response (“sufficient”).

In addition, over 77% of nurses disagreed with the subjective statement: “The role of registered nurses in regard to care of patients with TBI is clearly understood in my workplace.” This finding emphasizes there may be an underlying issue with role clarity or perception of role clarity; to address this underlying issue, additional education and training is needed, particularly as it relates to the nursing role in caring for patients with TBI. However, this finding may be influenced by the low frequency of caring for patients with moderate-to-severe TBI at the recruitment hospitals. A positive finding was that over 80% of nurses correctly agreed with the statement, “Knowing about moderate-to-severe traumatic brain injury is important to my current nursing practice.” The high-level of agreement with this statement indicates that nurses value knowledge about moderate-to-severe TBI, perhaps because they feel it is relevant to their practice. This accurate belief is important to learning effective care practices for patients with moderate-to-severe TBI [41].

### Practice implications

Similar to other literature [16], [21], [22], [24], these findings emphasize the need for additional education and training for VHA nurses who care for patients with moderate-to-severe TBI. Coupled with the increasing incidence of Veterans receiving care at VHA hospitals across the nation [1], [12], these findings suggest the need for: 1) specialized education and training for nurses who care for patients with moderate-to-severe TBI and 2) evidence-based practice guidelines for nurses to use as a resource for non-acute nursing management of these patients. The specialized and often complex medical conditions that are typically seen in patients with moderate-to-severe TBI require healthcare providers, particularly nurses, to have specialized knowledge [42]. This knowledge can be imperative to achieving and improving patient outcomes [42]. In addition, our findings show little to no TBI-related content was covered in nursing school curriculum, as only 7% of nurses in our sample with TBI-related training received the training as part of their undergraduate or graduate school education. These findings emphasize that TBI-related training may need to be provided as on-the-job continuing education or training specific to the nursing role (e.g., nursing assessment, diagnosis, planning, implementation, and evaluation) because detailed information about caring for patients with TBI is not typically included in undergraduate and graduate nursing school curriculum. Finally, although our findings focus on Veteran patients with moderate-to-severe TBI, there are high rates of Veteran patients with mild TBI. Symptoms of mild TBI are more difficult to recognize and may be dismissed with the assumption that the patient should have recovered. However, mild TBI symptoms also have an influence on the nursing plan of care, which warrants future exploration of nurses’ perceptions of caring for Veteran patients with mild TBI.

### Limitations

We collected data from VHA nurses across departments instead of targeting nurses who care for Veteran patients with moderate-to-severe TBI on a regular basis. However, findings showed it would be difficult to target nurses who cared for Veteran patients with TBI regularly without recruiting from several VHA hospitals as low frequencies of care of patients with TBI were reported in the sample (see Table 2) and the Polytrauma System of Care classification varies from one VHA hospital to the next. Thus, the nature of the sample may limit generalizability. Although our findings may not be fully representative of the TBI-related training and beliefs of nurses who care for patients with moderate-to-severe TBI on a frequent basis, these findings provide a foundation for future research. Second, we did not have any items that differentiated between patients with acute or chronic moderate-to-severe TBI; instead, we chose to have items that generally referred to “patients with moderate-to-severe TBI” for ease of data collection. Not differentiating between acute and chronic moderate-to-severe TBI may have decreased nurses’ reports of experience practicing with these patients or frequency of care. However, findings showed almost 90% of nurses have ever cared for patients with TBI, so lack of differentiation did not seem to affect our findings.

### Future research

Future researchers may choose to compare the training and beliefs of nurses who care for Veteran patients with moderate-to-severe TBI on a frequent basis to those who do not. Similarly, additional studies may be needed to determine the TBI-related training and beliefs of nurses who frequently care for Veteran patients with acute moderate-to-severe TBI compared to nurses who frequently care for Veteran patients with chronic moderate-to-severe TBI, as experience working with each TBI sub-population may influence training completed or beliefs held.

## Conclusion

The increasing incidence rates of Veteran patients with moderate-to-severe TBI and the fact that Veterans seek care at all hospitals, not just VHA hospitals, emphasizes the need for all nurses to be knowledgeable about care of these patients. Although there were a number of accurate beliefs in the sample, these findings suggest there are areas where additional training may be needed. Areas for improvement included: 1) further clarity in the role of a nurse when caring for a Veteran patient with moderate-to-severe TBI, 2) behavior manifestations in Veteran patients with moderate-to-severe TBI; and 3) nursing care plan development for Veteran patients with moderate-to-severe TBI.

The PBIS can be used to determine differences in the amount and type of TBI-related training and inaccuracies in beliefs, which can provide insight on nurses’ preparation to care for this patient population. In addition, these findings can be used to direct development and testing of appropriate educational and training interventions, which can ensure nurses receive the knowledge and training needed to provide effective and consistent care to improve patient outcomes of patients with TBI.

## Supporting information

S1 STROBE Checklist (DOC)
